# Molecular and Conventional Analysis of Acute Diarrheal Isolates Identifies Epidemiological Trends, Antibiotic Resistance and Virulence Profiles of Common Enteropathogens in Shanghai

**DOI:** 10.3389/fmicb.2018.00164

**Published:** 2018-02-09

**Authors:** Feng Yang, Yonggen Jiang, Lihua Yang, Juanxiu Qin, Mingquan Guo, Yuxia Lu, Hongyou Chen, Yuan Zhuang, Jinghao Zhang, Hong Zhang, Zhaoyun Dai, Min Li, Changqing Yang, Min Chen, Yanmei Zhang, Hu Zhao

**Affiliations:** ^1^Department of Laboratory Medicine, Huadong Hospital Affiliated to Fudan University, Shanghai, China; ^2^Shanghai Key Laboratory of Clinical Geriatric Medicine, Shanghai, China; ^3^Research Center on Aging and Medicine, Fudan University, Shanghai, China; ^4^Songjiang Center for Disease Control and Prevention, Shanghai, China; ^5^Department of Laboratory Medicine, Renji Hospital, School of Medicine, Shanghai Jiao Tong University, Shanghai, China; ^6^Department of Gastroenterology, Tongji Hospital Affiliated to Tongji University, Shanghai, China; ^7^Shanghai Municipal Center for Disease Control and Prevention, Shanghai, China; ^8^Department of Laboratory Medicine, Children’s Hospital of Shanghai, School of Medicine, Shanghai Jiao Tong University, Shanghai, China; ^9^Department of Infectious Diseases, Huadong Hospital Affiliated to Fudan University, Shanghai, China

**Keywords:** acute diarrhea, epidemiology, pathogens, resistance, multilocus sequence type (MLST), virulence

## Abstract

**Objective:** To investigate prevalence of acute diarrhea in Shanghai and analyze virulence associated-genes and antibiotic resistance of major enteropathogens using combination of conventional and molecular epidemiology methods.

**Method:** The 412 stool specimens were obtained by systematic sampling from diarrhea patients throughout entire year 2016. Bacterial and viral pathogens were identified and bacterial isolates were cultured and screened for antibiotic resistance profiles. Two most prevalent bacteria, *Vibrio parahaemolyticus* and *Salmonella* were further typed by multi-locus sequence typing (MLST) and analyzed for presence of virulence-associated genes. The association between virulence genes, resistance phenotypes and genetic diversities was analyzed.

**Results:** Among stool specimens testing positive for pathogens (23.1%), 59 bacterial and 36 viral pathogens were identified. *V. parahaemolyticus* (27/412, 6.6%), *Salmonella* (23/412, 5.6%) and norovirus GII (21/412, 5.1%) were three most-commonly found. Most bacterial isolates exhibited high levels of antibiotic resistance with high percentage of MDR. The drug resistance rates of *V. parahaemolyticus* and *Salmonella* isolates to cephalosporins were high, such as 100.0 and 34.8% to CFX, 55.6 and 43.4% to CTX, 92.6 and 95.7% to CXM, respectively. The most common resistance combination of *V. parahaemolyticus* and *Salmonella* was cephalosporins and quinolone. The dominant sequence types (STs) of *V. parahaemolyticus* and *Salmonella* were ST3 (70.4%) and ST11 (43.5%), respectively. The detection rates of virulence genes in *V. parahaemolyticus* were *tlh* (100%) and *tdh* (92.6%), without *trh* and *ureR*. Most of the *Salmonella* isolates were positive for the *Salmonella* pathogenicity islands (SPIs) genes (87–100%), and some for *Salmonella* plasmid virulence (SPV) genes (34.8% for *spvA* and *spvB*, 43.5% for *spvC*). In addition, just like the drug resistance, virulence genes exhibited wide-spread distribution among the different STs albeit with some detectable frequency linkage among *Salmonella* STs.

**Conclusion:** Bacterial infections are still the major cause of severe diarrheas in Shanghai. The most common bacteria *V. parahaemolyticus* and *Salmonella* show molecular characteristics consistent with preselection of highly virulent types with exceedingly high level of antibiotic resistance.

## Introduction

Diarrheal diseases are serious global public health problem (Guerrant et al., 2002). The World Health Organization’s Global Burden of Disease study reported that diarrheal diseases were the seventh leading cause of death in 2010, worldwide (Lozano et al., 2012). In 2015, diarrheal diseases caused globally 1.32 million deaths (GBD 2015 Mortality and Causes of Death Collaborators, 2016). In China, about 700000 cases of diarrheal diseases are reported annually (Zhu et al., 2008). Among the diarrheal diseases, acute diarrhea occurs most commonly and has most serious impact on human health in terms of mortality and medical costs (Das and Bhutta, 2016; Riddle et al., 2016).

Most of the causative agents of acute diarrheas are bacteria and viruses, among which *Salmonella*, *Shigella*, *Vibrio parahaemolyticus*, rotavirus, and norovirus account for a largest proportion of cases worldwide (Farthing et al., 2013). Studies report that the prevalence and composition of diarrheal pathogens varies from region to region. For example, rotavirus and diarrheagenic *Escherichia coil* (DEC) were the leading pathogens in Bangladesh in 2011 based on culture, polyacrylamide gel electrophoresis (PAGE) and Multiplex polymerase chain reaction (PCR) detections (Kabir et al., 2012). Rotavirus and *Shigella* were the main cause of diarrhea in western Kenya from 2009 to 2011 by culture and enzyme immunoassay (Swierczewski et al., 2013), while norovirus and *Clostridium difficile* were the most commonly detected pathogens in Australia in 2007 by culture, ELISA, reverse-transcription PCR (RT-PCR) and immunochromatography (Huhulescu et al., 2009). Calicvirus, rotavirus and *Campylobacter jejuni* were the most commonly detected pathogens in a Nordic country epidemiological survey from 2003 to 2007 by culture, RT-PCR and immunochromatography (Hilmarsdottir et al., 2012), while norovirus and *Salmonella* were the major pathogens for foodborne illness in the United States from 2000 to 2008 reported in a data estimate research (Scallan et al., 2011). However, the detailed etiological reports of acute diarrhea in a specific area are rare and these studies should be conducted to ensure proper epidemiological control and effective treatment, especially in densely populated areas. Shanghai is the largest urban agglomeration, which encompasses over 50 million people living at the highest population density in the world and experiencing huge amount of national and international traffic. From this perspective epidemiological surveillance of this area is particularly important to the global health.

Increasing occurrence of pathogenic *V. parahaemolyticus* and *Salmonella* promoted studies dissecting genetic typing of these pathogens and associations between their virulence factors and their pathogenicity (Scallan et al., 2011). The pathogenesis of *V. parahaemolyticus* is mainly attributed to the presence of thermostable direct hemolysin (TDH), thermolabile hemolysin (TLH) and TDH-related hemolysin (TRH), which are encoded by the *tdh*, *tlh*, and *trh* genes, respectively (Taniguchi et al., 1986). The pathogenicity of *Salmonella* is linked to a variety of virulence factors present in the chromosomal *Salmonella* pathogenicity islands (SPIs) and the *Salmonella* plasmid virulence (SPV) operon (Groisman and Ochman, 1996, 1997). Except for bacteria gastroenteritis, viral gastroenteritis is also one of the most common illnesses in human worldwide (Mo et al., 2015). Previous studies showed that the pathogenesis of viruses was due to viral genome types and environmental factors (Aly et al., 2007; Vidovic et al., 2011). Thus, analysis of the specific-virulence related genes is needed for better understanding the mechanisms of pathogenicity and to predict possible risks and outcomes of infection with various substrains.

Since, bacterial pathogens are the major cause of diarrheal diseases, ever-increasing antibiotic resistance is one of the most serious consideration in choice for their treatment (Arias and Murray, 2009). Thus analysis of antibiotic resistance is the crucial component of bacteriological diagnostics. Epidemiological studies of several hospitals in different cities of China revealed that resistance especially, multi-drug or pan-drug resistance kept increasing with the alarming rate (Gong et al., 2017; He et al., 2017; Hu et al., 2017; Yang et al., 2017). Thus, the investigation of antibiotic resistance spectrum of diarrheal bacteria may provide important information for healthcare providers in proper treatments and taking measures to reduce drug resistance of bacteria.

Finally, studies increasingly demonstrated the associations between most prevalent genotypes with particular virulence attributes and types of antimicrobial drug resistance in the clinical bacterial isolates. Such relationships were reported in *Listeria monocytogenes*, *Klebsiella pneumoniae*, *V. parahaemolyticus* and *Salmonella* (Campioni et al., 2012; Luo et al., 2014; Wu et al., 2016; Li et al., 2017). However, the study of genetic diversity of enteric bacterial pathogens has become a great challenge due to the high rates of recombination and mutation resulting from rapid selection and microevolution of prevalent microbial sub-strains (Denamur and Matic, 2006). Compared to other typing methods, including serotyping and pulsed-field gel electrophoresis (PFGE), MLST was proposed to provide one of the most effective tools for investigating the prevalence and genetic diversity of pathogenic bacteria at multiple levels (Achtman et al., 2012; Jones et al., 2012).

The aim of our study was to analyze the clinical epidemiology of infectious acute diarrhea, explore the seasonality and distribution of the pathogens and to gain insight into antimicrobial resistance, prevalence of virulence genes and genetic diversities. Considering Shanghai is one of the greatest international metropolis in the world with a huge flow of people, the study of the epidemiological characteristics and pathogen features of acute diarrhea have important local and global implications in understanding pathogens epidemiology and evolution.

## Materials and Methods

### Ethics Statement

This study has been approved by Ethics Committee of Huadong Hospital. Verbal informed consent was obtained from every participant and all personal information was de-identified to protect patient privacy. The Ethics Approval Number: [2013-077].

### Inclusion and Exclusion Criteria

#### Inclusion Criteria

Both male and female, with symptoms of acute diarrhea were included, without age limitation. According to the American College of Gastroenterology (ACG) Clinical Guideline from World Gastroenterology Organization (WGO) (Riddle et al., 2016), diarrhea was defined as the defecation frequency three or more times per 24 h, with abnormal fecal characteristics, such as loose, watery, mucous or bloody stool. Acute diarrhea was defined as the course of disease lasted for less than 14 days.

#### Exclusion Criteria

Diarrhea caused by medicine, poison, food allergy or food intolerance; diarrhea caused by other gastrointestinal diseases; diarrhea caused by irritable bowel syndrome (IBS); persistent diarrhea (lasting for more than 14 days); systemic use of antibiotics after diarrhea.

### Clinical Specimen Collection

All patients eligible for enrollment in the Center Hospital of Songjiang District were numbered according to the registration order from January to December in 2016, and one from each five patients was selected into the research by systematic sampling. Stool specimens were collected from each patient at the time of enrollment. For viral testing, fresh whole stool was collected in sterilized containers with normal saline and RNase inhibitor and stored at -20°C for further detection. For bacterial testing, fresh whole stool was inoculated onto *Salmonella-Shigella* (SS) agar, Columbia blood agar, charcoal cefoperazone deoxycholate agar (CCDA) and selective enrichment broth (3% NaCl alkaline peptone water) within 2 h. Each participant’s clinical characteristics, epidemiological information and laboratory results were recorded using a standardized patient reporting form.

### Nucleic Acid Extraction and Sequencing

Total nucleic acid was extracted from 200 μL of 10% fecal suspension prepared in normal saline using the QIAsymphony Virus/Bacteria Mini kit (Qiagen, United Kingdom) according to the QIAsymphony SP instrument (Qiagen, United Kingdom). Genomic DNA of the isolates was extracted using the TIANamp bacteria DNA Kit (Tiangen Biotech Beijing Co., Ltd, Beijing, China). The extract was eluted in 50 μL DNase-free and RNase-free water and stored at -80°C. The quality of the DNA extract was assessed using a NanoDrop spectrophotometer (ND-1000; Thermo Fisher Scientific Inc., Waltham, MA, United States). All procedures were conducted following the manufacturers’ instructions. PCR products were purified using the high pure PCR product purification kit (Roche, Basel, Switzerland) according to the manufacturer’s instructions. The sequencing was performed by Sanger method using the ABI 3730XL automated DNA analyzer (Applied Biosystems Inc., Foster City, CA, United States). The information of primers for sequencing was listed in the Supplementary Table 1. The DNA sequence analysis was performed using the DNAStar Lasergene software (DNAstar Inc., Madison, WI, United States).

### Bacterial Culture and Identification

Stool specimens were inoculated on SS agar and Columbia blood agar at 37°C for 24 h; CCDA under micro-aerophilic culture at 37°C for 24 h; selective enrichment broth at 37°C for 18–24 h, followed by subculture on thiosulphate citrate bile salt sucrose (TCBS) agar at 37°C for 24 h. According to the 11th Manual of Clinical Microbiology in 2015 (Jorgensen and Pfaller, 2015), colonies of suspected *Salmonella* and *Shigella* (with colorless transparent or black colonies on SS agar) were identified by VITEK-2 Compact (BioMerieÌux, Lyon, France) and serotyping (SSI Diagnostica, Hillerod, Denmark), colonies of suspected *C. jejuni* (with gray-white colonies and about one millimeter in diameter on CCDA agar), *Vibrio cholerae* (with yellow colonies on TCBS agar) and *V. parahaemolyticus* (with blue-green colonies on TCBS agar) were identified by Matrix-Assisted Laser Desorption Ionization time-of-flight mass spectrometry (BioMerieÌux, Lyon, France). *V. parahaemolyticus* was further analyzed by serotyping using respective antisera (Denka Seiken CO., LTD., Japan).

### Antimicrobial Susceptibility Testing

The antibiotic sensitivities of all isolates were tested by the standard disk diffusion method according to the Clinical and Laboratory Standards Institute (CLSI) guidelines (2016), using Mueller Hinton agar (Chromagar, Paris, France) to culture *Salmonella*, *Shigella* and *V. parahaemolyticus* and Mueller Hinton agar medium with 5% sheep blood to culture *C. jejuni*. Resistance spectra for *Salmonella*, *Shigella* and *V. parahaemolyticus* were established based on susceptibility to 11 antimicrobials belonging to six classes: β-lactam (cefoxitin: CFX, cefotaxime: CTX, cefuroxime: CXM), quinolone (ciprofloxacin: CIP, levofloxacin: LVX, norfloxacin: NOR, nalidixic acid: NAL), aminoglycoside (gentamicin: GEN), tetracycline (tetracycline: TCY), sulfonamides (compound sulfamethoxazole: CO-SMZ) and chloramphenicol (chloramphenicol: CHL). Four antibiotics reported in CLSI guidelines were used to test the antibiotic susceptibilities of *C. jejuni*, which belong to three classes: macrolides (erythromycin: ERY), quinolone (CIP) and tetracycline (TCY, doxycycline: DOX). Multidrug-resistant (MDR) was defined as acquired non-susceptibility to at least one agent in three or more antimicrobial categories (Magiorakos et al., 2012). *E. coli* ATCC 25922 was used as the quality control bacterium for the antimicrobial susceptibility testing.

### Virus Detection

Quantitative PCR was used to analyze total nucleic acid samples for detection of five viruses, including: norovirus, sapovirus, rotavirus, astrovirus and adenovirus using their respective real-time PCR kits (BioPerfectus Technologies, Taizhou, China) in the Applied Biosystems real-time PCR system (7500 real-time PCR system, ABI, Foster City, CA, United States) with 7500 system software v2.3. The reaction conditions for the amplifications were as follows: 95°C for 5 min, followed by 45 cycles of denaturation at 95°C for 10 s, annealing, extension and fluorescence detection at 55°C for 40 s. All the procedures were conducted according to the instructions.

### Detection of Virulence-Associated Genes

*Vibrio parahaemolyticus* isolates were tested for the presence of virulence genes *tlh, tdh*, *ureR*, and *trh. Salmonella* isolates were tested for the presence of virulence genes SPI-1(*invA*, *hilA*), SPI-2 (*ssaQ*, *ssrB*, *spiA*), SPI-3 (*mgtC*), SPI-4 (*orfL*), SPI-5 (*sopB*), SPI-6 (*safB*), *spvA*, *spvB* and *spvC*. The virulence genes of each isolate were amplified individually using Taq PCR MasterMix (Tiangen Biotech Co., Ltd.) on a GeneAmp 9700 thermal cycler (Applied Biosystems, Foster City, NY, United States). DNase-free and RNase-free water was used as negative control and 16S rDNA gene as positive control. The specific primers were listed in Supplementary Table 1. The PCR fragments were analyzed by agarose gel electrophoresis.

### MLST Analysis

*Vibrio parahaemolyticus* and *Salmonella* isolates were analyzed using MLST to detect the following seven housekeeping genes: *recA*, *dnaE*, *gyrB*, *dtdS*, *pntA*, *pyrC* and *tnaA* for *V. parahaemolyticus*; *thrA*, *purE*, *sucA*, *hisD*, *aroC*, *hemD* and *dnaN* for *Salmonella*. MLST were carried out in accordance with the criteria published in the corresponding MLST database websites (^1^ for *V. parahaemolyticus*;^2^ for *Salmonella*). The sequences were compared and identified with the existing sequences available from the MLST websites. The information of the primers was listed in Supplementary Table 1. The minimal spanning trees (MST) were generated using BioNumerics 7.5 (Applied Maths, Belgium).

### Statistics

All statistical analyses were performed using Stata/SE 14.0 (Stata Corp, College Station, TX, United States) for Mac. Differences between the groups were compared by Chi-square tests or Person Chi-square tests. Statistical significance was defined at a *P* < 0.05.

## Results

### Clinical Characteristics and Isolation Rates of Enrolled Patients with Acute Diarrhea

A total of 412 patients (204 females and 208 males) with acute diarrhea were selected out of 2059 outpatients by systematic sampling in 2016 upon application of strict inclusion and exclusion criteria (**Table 1**). Most cases of acute diarrhea occurred in summer (39.3%) and autumn (26.7%), and fewer in spring (17.5%) and winter (16.5%). The median age was 37 years [Interquartile Range (IQR): 28–56 years] and the median frequency of diarrheal episodes was 6 times per day (IQR: 4-8 times). Watery stool was the most common symptomatic characteristics (59.7%), followed by loose stool (28.6%), mucous stool (10.0%) and bloody stool (1.7%).

**Table 1:** Clinical characteristics and isolation rates of enrolled patients with acute diarrhea.

Groups		Number of cases	Constituent ratio (%)	Number of pathogens	Isolation rate (%)
Gender	Male	208	50.5	48	23.1
	Female	204	49.5	47	23.0
Age(year)	0–19	25	6.1	4	16.0
	20–39	199	48.3	43	21.6
	40–59	108	26.2	26	24.1
	60–79	72	17.5	19	26.4
	≥80	8	1.9	3	37.5
Characteristic	Loose stools	118	28.6	20	16.9
	Watery stools	246	59.7	67	27.2
	Mucus in stools	41	10.0	7	17.1
	Blood in stools	7	1.7	1	14.3


### Major Acute Diarrheal Pathogens Were Confirmed in Stool Specimens

**FIGURE 1:**
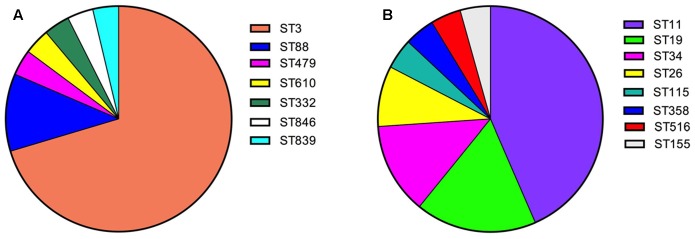
Sequence types (STs) of the *V. Parahaemolyticus* and *Salmonella* diarrheal isolates. The 412 stool specimens were obtained by systematic sampling from diarrhea patients in Shanghai throughout entire year 2016. STs of 27 *V. parahaemolyticus* isolates **(A)**, and of 23 *Salmonella* isolates detected in this study **(B)** were analyzed and their relative frequencies within each species calculated.

**FIGURE 2:**
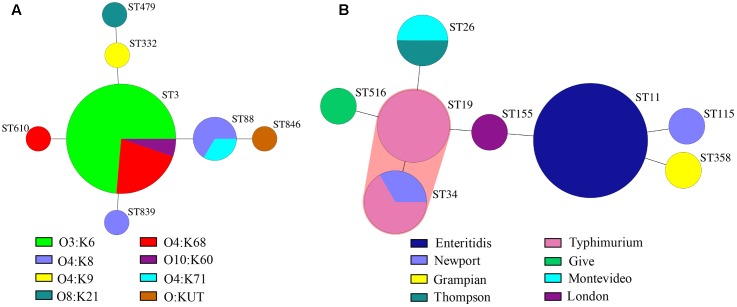
Minimal Spanning Trees of the *V. parahaemolyticus and Salmonella* isolates. Minimal spanning tree (MST) generated from MLST data of *V. parahaemolyticus* **(A)** and *Salmonella* **(B)** isolates. The color indicates the serotypes of isolates. The sizes of the nodes represent the numbers of isolates of each ST. The distance (connecting lines) between STs depict the number of allelic differences between them. Note that the MST analysis does not show much clustering patterns.

Infectious diarrhea (with confirmed etiological cause) occurred in 93 out of 412 patients. A total of 95 pathogens including 59 bacteria and 36 viruses were identified in these stool specimens (**Table 2**). The single pathogen infection was observed in 91 cases and polymicrobial infection in two cases. Viral pathogens were detected in 8.7% of all the specimens. Norovirus was the most common virus and the detection rate was 5.3%. The GII was the predominant Norovirus type detected, accounting for 95.5% of all cases, including one case of norovirus GII and GI co-infection. Eight cases of sapoviruses (1.9%) and four cases of rotavirus infection (1.0%) were also confirmed. All four rotavirus enteritis cases were caused by Group A rotavirus. Bacterial pathogens were identified in 14.3% of all the patients, and the two predominant bacteria were *V. parahaemolyticus* (6.6%) and *Salmonella* (5.6%), including one case of *V. parahaemolyticus* and *Salmonella* co-infection. There was no significant difference in the positive detections among age groups (*p* = 0.664), gender (*p* = 0.993) and other aspects of patient clinical characteristics (*p* = 0.110) (**Table 2**). The two dominant pathogen *V. parahaemolyticus* and *Salmonella* isolates were further analyzed by MLST and serotyping. MLST classified these 27 *V. parahaemolyticus* isolates into seven STs with high genetic diversity (**Figure 1A**). ST3 was the most commonly found isolate type of *V. parahaemolyticus* (70.4%), followed by ST88 (11.1%). Individual cases of infections (amounting to 3.7%) were represented by ST332, ST479, ST610, ST839 and ST846. MST analysis of *V. parahaemolyticus* isolates mainly showed the distinct but not apparent clustering patterns (**Figure 2A**). The predominant serotype of *V. parahaemolyticus* was O3:K6 (51.9%) (O: somatic antigen; K: capsular antigen), followed by O4:K68 (18.5%) and O4:K8 (11.1%) (**Table 3**). Remarkably, all O3:K6 isolates were ST3. For the 23 *Salmonella* isolates, eight distinct STs were identified (**Figure 1B**), and the ST11 (43.5%) was the most frequently detected type, followed by ST19 (17.4%), ST34 (13.0%) and ST26 (8.7%). Other detected STs: ST115, ST155, ST358 and ST516 represented individual cases (4.3%). Furthermore, MST analysis of *Salmonella* isolates showed ST19 and ST34 clustered in the same complex (**Figure 2B**). Besides, *Salmonella* isolates were classified into eight serotypes, the dominant types were *Salmonella enteritidis* (43.5%) and *Salmonella typhimurium* (26.1%). All *Salmonella enteritidis* isolates were ST11, all *Salmonella typhimurium* isolates were ST19 or ST34.

**Table 2:** Distribution of pathogens in stool samples varied from patients with acute diarrhea.

Pathogens	Positive numbers (*n*[%]) (*n* = 412)	Age Groups (*n*[%])					Gender (*n*[%])		Stool characteristics (*n*[%])			
				
		<20y	20–39y	40–59y	60–79y	≥ 80y	Male	Female	Loose	Watery	Mucus	Bloody
		(*n* = 25)	(*n* = 199)	(*n* = 108)	(*n* = 72)	(*n* = 8)	(*n* = 208)	(*n* = 204)	(*n* = 118)	(*n* = 246)	(*n* = 41)	(*n* = 7)
***Bacteria***	59 (14.3)	2 (8.0)	30 (15.1)	14 (13.0)	11 (15.3)	2 (25)	27 (13.0)	32 (15.7)	14 (11.9)	39 (15.9)	6 (14.6)	0 (0)
*Salmonella*	23 (5.6)	0 (0)	10 (5.0)	7 (6.5)	5 (6.9)	1 (12.5)	11 (5.3)	12 (5.9)	8 (6.8)	13 (5.3)	2 (4.9)	0 (0)
*Shigella*	5 (1.2)	1 (4.0)	3 (1.5)	0 (0)	0 (0)	1 (12.5)	2 (1.0)	3 (1.5)	0 (0)	3 (1.2)	2 (4.9)	0 (0)
*V. parahaemolyticus*	27 (6.6)	1 (4.0)	15 (7.5)	5 (4.6)	6 (8.3)	0 (0)	13 (6.3)	14 (6.9)	4 (3.4)	21 (8.5)	2 (4.9)	0 (0)
*V. cholerae*	0 (0)	0 (0)	0 (0)	0 (0)	0 (0)	0 (0)	0 (0)	0 (0)	0 (0)	0 (0)	0 (0)	0 (0)
*C. jejuni*	4 (1.0)	0 (0)	2 (1.0)	2 (1.9)	0 (0)	0 (0)	1 (0.5)	3 (1.5)	2 (1.7)	2 (0.8)	0 (0)	0 (0)
**Viruses**	36 (8.7)	2 (8.0)	13 (6.5)	12 (11.1)	8 (11.1)	1 (12.5)	21 (10.1)	15 (7.4)	6 (5.1)	28 (11.4)	1 (2.4)	1 (14.3)
Norovirus (GI, GII)	22 (5.3)	2 (8.0)	11 (5.5)	5 (4.6)	3 (4.2)	1 (12.5)	15 (7.2)	7 (3.4)	3 (2.5)	17 (6.9)	1 (2.4)	1 (14.3)
Sapovirus	8 (1.9)	0 (0)	1 (0.5)	3 (2.8)	4 (5.6)	0 (0)	4 (1.9)	4 (2.0)	1 (0.8)	7 (2.8)	0 (0)	0 (0)
Rotavirus	4 (1.0)	0 (0)	0 (0)	3 (2.8)	1 (1.4)	0 (0)	1 (0.5)	3 (1.5)	1 (0.8)	3 (1.2)	0 (0)	0 (0)
Astrovirus	2 (0.5)	0 (0)	1 (0.5)	1 (0.9)	0 (0)	0 (0)	1 (0.5)	1 (0.5)	1 (0.8)	1 (0.4)	0 (0)	0 (0)
Adenovirus	0 (0)	0 (0)	0 (0)	0 (0)	0 (0)	0 (0)	0 (0)	0 (0)	0 (0)	0 (0)	0 (0)	0 (0)
**Total**	**95 (23.1)**	**4 (16)**	**43 (21.6)**	**26 (24.1)**	**19 (26.4)**	**3 (37.5)**	**48 (23.1)**	**47 (23.0)**	**20 (16.9)**	**67 (27.2)**	**7 (17.1)**	**1 (14.3)**


**Table 3:** The association of virulence genes and resistance phenotype with genetic diversity and serotypes of the *V. parahaemolyticus* and *Salmonella* isolates.

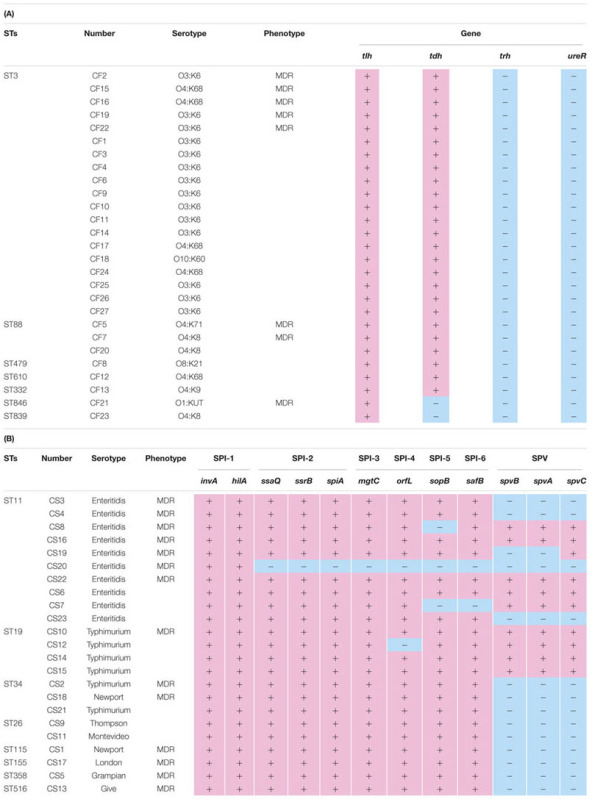

**Summary of virulence genes, resistance phenotype and genetic types (ST), serotypes of *V. parahaemolyticus***(A)** and *Salmonella***(B)** isolates was conducted. Presence (magenta) or absence (blue) of four virulence-associated genes of each *V. parahaemolyticus* and twelve virulence-associated genes of *Salmonella* including six SPIs and SPV, was established by PCR sequencing for each isolated organism. Note that there was no statistical difference in the MDR rates among different STs and serotypes. O, somatic antigen; K, capsular antigen; KUT, K antigen un-typeable.**

### Acute Diarrheal Pathogens Presented Differential Seasonal Distribution

**FIGURE 3:**
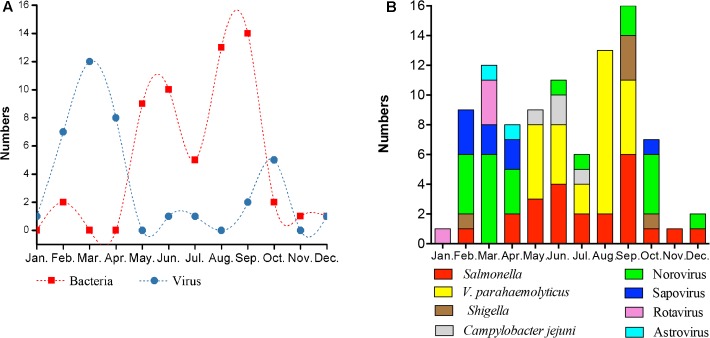
Acute diarrheal pathogens presented seasonal distribution. Numbers of cases for bacterial (red) and viral (blue) diarrheal infections confirmed by laboratory detections of pathogens in stool samples summarized on monthly basis **(A)**. Note distinct, non-overlapping peaks of occurrence of bacterial versus viral infections. Cumulative, monthly detections of individual bacterial and viral pathogens throughout year 2016 are shown on **(B)**.

Our findings indicated that the incidence of infections with specific pathogens showed very strong seasonal trends and different groups of pathogens showed distinct distribution patterns (**Figure 3**). Bacterial pathogens were more frequently detected in summer and early autumn, while viral pathogens were more commonly detected in winter and spring (**Figure 3A**). The bacterial infections were prevalent during hot months of the year (**Figure 3B**) but each of the bacterial species causing infections followed its own specific seasonal distribution. *V. parahaemolyticus* was detected from May to September, peaking in August, while *C. jejuni* was causing infections only in May through July. In contrast *Salmonella* was detected in most months of the year, with some increase in September. The peak of viral infections was in March, both with respect to overall number of cases and diversity of viruses causing infections. However, Norovirus was detected in more than half of the months of the year while Rotavirus, Astrovirus, and Sapovirus infections were mostly found only in late winter/spring.

### Bacterial Pathogens Associated with Acute Diarrhea Showed High Level of Antimicrobial Resistance

**FIGURE 4:**
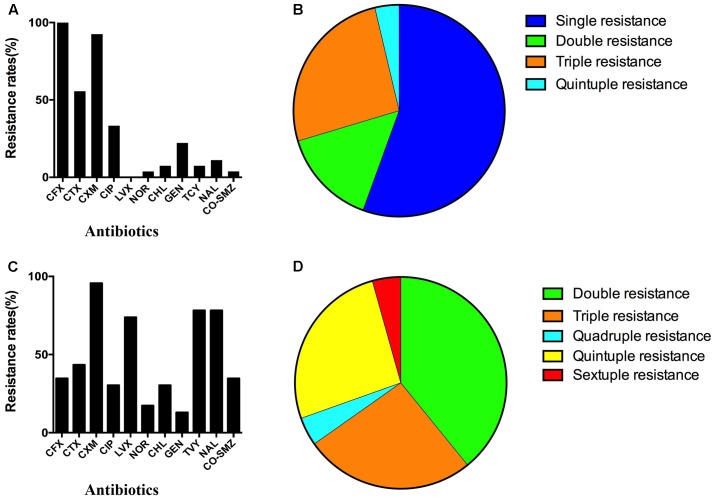
Resistance profiles of the *V. parahaemolyticus* and *Salmonella* isolates to different antibiotics. For each culture isolate of bacterial EP antibiotic resistance was evaluated using the standard disk diffusion method. Resistance rates of *V. parahaemolyticus* isolates to 11 antibiotics and drug resistance profile of *V. parahaemolyticus* isolates are shown in **(A,B)**, respectively. Resistance rates of 23 *Salmonella* isolates to 11 antibiotics and drug resistance profile of *Salmonella* isolates are shown in **(C,D)**. CFX, cefoxitin; CTX, cefotaxime; CXM, cefuroxime; CIP, ciprofloxacin; LVX, levofloxacin; NOR, norfloxacin; CHL, chloramphenicol; GEN, gentamicin; TCY, tetracycline; NAL, nalidixic acid; CO-SMZ: compound sulfamethoxazole.

The resistance against antimicrobial agents was high among all bacterial pathogens (**Figure 4**). All of the clinical *V. parahaemolyticus* isolates exhibited high resistance rates to cephalosporins, including second-generation cephalosporins cefoxitin (CFX, 100%), cefuroxime (CXM, 92.6%) and third-generation cephalosporins cefotaxime (CTX, 55.6%) (**Figure 4A**). The resistance rates of *V. parahaemolyticus* isolates to quinolone (CIP) and gentamicin (GEN) were 33.3 and 22.2%, respectively. By contrast, *V. parahaemolyticus* showed low resistance rates to levofloxacin (LVX, 0), norfloxacin (NOR, 3.7%), chloramphenicol (CHL, 7.4%), tetracycline (TCY, 7.4%), nalidixic acid (NAL, 11.1%) and compound sulfamethoxazole (CO-SMZ, 3.7%). In addition, 65.1% *V. parahaemolyticus* isolates were resistant to one antibiotic class, all of which belong to cephalosporins; 14.8% isolates were resistant to two classes of antibiotic, cephalosporins combined with quinolone; 25.9% isolates were resistant to three classes of antibiotic, 85.7% of which were cephalosporins, quinolone combined with aminoglycoside; 3.7% isolates were resistant to four classes of antibiotic (**Figure 3B**). Finally, 29.6% *V. parahaemolyticus* isolates were MDR.

The drug resistance rates of *Salmonella* to cephalosporins were also high, including CXM (95.7%), CTX (43.4%) and CFX (34.8%) (**Figure 4C**). In addition, 78.3% of *Salmonella* isolates were resistant to TCY and NAL, and 73.9% were resistant to LVX. *Salmonella* isolates also exhibited resistance to CIP (30.4%), NOR (17.4%), CHL (30.4%), GEN (13.0%) and CO-SMZ (34.8%). Besides, 39.1% *Salmonella* isolates were double resistance to cephalosporins combined with quinolone; 26.1% isolates were triple resistance, among which the most common combination (83.3%) were cephalosporins, quinolone and tetracycline; 4.3% isolates were resistant to four classes of antibiotic; 26.1% isolates were resistant to five classes of antibiotic except for aminoglycoside; 4.3% isolates were resistant to all six classes of antibiotic. Altogether, the results showed that 60.9% *Salmonella* isolates were MDR (**Figure 4D**).

Among all five *Shigella* isolates, four were resistant to GEN, TCY, NAL, CO-SMZ, CTX, CXM and CIP, and one was resistant to GEN, TCY, NAL and CO-SMZ, and thus the MDR rate for *Shigella* isolates in this study was 100%. All four *C. jejuni* isolates were resistant to TCY, three were resistant to CIP, and two were resistant to erythromycin (ERY) and doxycycline (DOX). Two MDR *C. jejuni* isolates were detected in this study.

### Distribution of Virulence-Associated Genes of the Dominant Bacterial Pathogens

To analyze the pathogenicity of *V. parahaemolyticus*, four known virulence-associated genes including *tlh*, *tdh*, *trh* and *ureR* were detected by PCR sequencing (**Table 3**). The *tlh* gene was detected in all *V. parahaemolyticus* isolates, and *tdh* gene was detected in most of the isolates (92.6%). Interestingly, variable hemolysin gene *trh* was undetected in all the isolates, and *ureR* gene expected to be found upstream of *trh* in *V. parahaemolyticus* genome was also undetected.

Twelve virulence genes including nine genes located in six SPIs and three SPV genes were selected to screen for pathogenicity of *Salmonella*. SPI-1 genes *invA* and *hilA* were detected in all *Salmonella* isolates; 22 isolates (95.7%) were positive for the presence of SPI-2 genes (*ssaQ*, *ssrB* and *spiA*) and SPI-3 gene *mgtC* (**Table 3**). Furthermore, 21 isolates (91.3%) were positive for SPI-4 gene *orfL*, while 20 (87.0%) isolates were positive for SPI-5 gene *sopB* and 21 isolates (91.3%) were positive for SPI-6 gene *safB*. Notably, only nine isolates (34.8%) were positive for the plasmid genes *spvA* and *spvB*, and ten isolates were positive for *spvC*.

### The Associations of Virulence Genes and Resistance Phenotypes with Serotypes and Genetic Diversities

The association of virulence with STs and serotypes of *V. parahaemolyticus* and *Salmonella* isolates was analyzed (**Table 3**). We found that *tlh* was detected in all STs, *tdh* in most, while *trh* and *ureR* were undetected in all STs of *V. parahaemolyticus* isolates, suggesting that *tlh* is the most important factor required for virulence and *thr* and *ureR* are dispensable. The predominant ST of *Salmonella* isolates was ST11, most of them posed the SPI genes (70.0–100.0%) and almost half of them posed SPV genes, while one isolate was found positive for only two SPI-1 genes, *invA* and *hilA* (**Table 3B**). Remarkably, almost all the virulence genes were detected among ST19 isolates (97.9%), while all SPI genes but none SPV genes were detected among ST34, ST26, ST115, ST155, ST358 and ST516 isolates. In term of the association with serotypes, the virulence genes posed in S*almonella enteritidis* were totally consistent with ST11 isolates, while *Salmonella typhimurium* isolates were positive for most SPI genes (98.1%) and part of SPV genes (66.7%), which was different with ST19 and ST34 isolates.

Regarding association of antibiotic resistance with STs and serotypes (**Table 3**), there was not any major resistance patterns associated with specific ST and serotypes in *V. parahaemolyticus* isolates. In ST3 *V. parahaemolyticus*, 30% of isolates were MDR and this frequency was similar in ST88, however the ST88 was only represented by three isolates. Interestingly, all the ST3 *V. Parahaemolyticus* isolates exhibited resistance to cefuroxime (CXM), which belongs to β-lactam antimicrobial agents. For *Salmonella* isolates, 68.4% of the ST11 (also *Salmonella enteritidis*) was MDR (**Table 3**). None apparent association between genetic diversities with its resistance phenotypes was detected in our results, consistent with uniformly wide-spread resistance among all most prevalent STs. In fact, no significant difference in the MDR rates among different STs and serotypes was found in both of *V. parahaemolyticus* and *Salmonella* isolates.

## Discussion

Shanghai is a “gateway to China” and the largest agglomeration worldwide experiencing huge amount of national and international traffic. From the epidemiological stand point updates on the epidemiological status need to be reported and recorded on regular basis. This in comparison with other major urban centers in the world will allow to better track the spread of GI infections and to observe local and global microevolution of GI pathogens. In this study, microbiological surveillance of 412 stool specimens from total of 2059 diarrhea patients has been conducted between January and December in 2016 in Shanghai. The enteric pathogens were confirmed via successful isolation in 93 out of the 412 stool specimens. Fifty-nine bacteria and thirty-six viruses were detected, among which *V. parahaemolyticus*, *Salmonella* and noroviruses were the leading etiologic agents. Among different pathogens many showed unique seasonal distribution, while others were common throughout the year. The two most common bacterial pathogens showed exceedingly high level of antimicrobial drug resistance. Finally, we found linkage of *V. parahaemolyticus* and *Salmonella* ST genetic variants and frequencies of specific virulence genes but not with drug resistance, further suggesting very wide spread of resistance genes across different unrelated strains of these pathogens.

The predominant enteric diarrheal pathogens detected in this study were *V. parahaemolyticus* (6.6%), *Salmonella* (5.6%) and Norovirus (5.3%). The isolation rate of *V. parahaemolyticus* was close to the 8.0% reported for southeastern China in previous years (Chen et al., 2016) and consistent with earlier report from Shanghai, in which *V. parahaemolyticus* was the most commonly detected bacterial pathogen (Zhang et al., 2014). However, Lei Jia’s study, which identified pathogens in almost 40% of patients in Beijing, reported calicivirus (16.1%) as the most prevalent pathogen, followed by rotavirus (11.3%), *V. parahaemolyticus* (5.1%) and *Salmonella* (3.9%) (Jia et al., 2016). *Salmonella* was the second most commonly detected EP in our studies and the isolation rate was 5.6%, which was higher than the previous study from Shanghai (0.9%) (Zhang et al., 2014) and Beijing (3.9%) (Jia et al., 2016), however, in all these studies *Salmonella* remained 2nd most common bacterial EP. Outside of China, comparative epidemiology shows even greater divergence. A Nordic country reported calicivirus, rotavirus and *Campylobacter jejuni* as the most common pathogens from 2003 to 2007 (Hilmarsdottir et al., 2012). Kenya reported rotavirus and *Shigella* as the main cause of diarrhea in 2009 to 2011 (Swierczewski et al., 2013). Australian report, ranks norovirus, followed by *C. difficile* and rotavirus as the most frequent causes of infectious diarrhea in 2007 (Huhulescu et al., 2009). These differences demonstrate that the pathogen spectrum significantly varies in different areas, the possible cause of these differences maybe the different races, diets and sanitation. However, these disparities were likely influenced by different research periods and variety of methods applied for detection. Thus, surveillance of GI pathogens implemented regularly at different study centers around the world will continue provide important updates regarding pathogen distribution.

Our results showed that in Shanghai norovirus remains the leading etiologic agent of viral diarrhea. Specifically, Norovirus GII accounted for 95.5% among the virus positive specimens in our study, consistent with the notion that GII viruses became established as the predominant type responsible for the majority of norovirus gastroenteritis in recent years worldwide (Estevez et al., 2013). Noroviruses cause nearly 200,000 deaths each year among children less than 5 years old in developing countries and efforts are being made to introduce preventative vaccines (Patel et al., 2009; Kim and Kim, 2014). Since, experimental norovirus vaccine studies show strong association between viral genotype and effectiveness of vaccine-induced protection (Atmar et al., 2011), our results support that the focus of vaccine development efforts should be on GII Norovirus group.

The analysis of seasonal distribution of etiological causes showed that there was strong seasonal dependence of enteric pathogen incidence. The epidemic peak of viral infections occurred mainly in winter/early spring whereas the peak of bacterial diarrheas occurred in summer and autumn, which was similar to the studies from other cities in China (Jia et al., 2016; Chi et al., 2017). Interestingly, peak of viral infection corresponded to lowest level of bacterial infections and vice versa, with the exception of November–January, during which incidences of both viral and bacterial infection levels were at their lowest points. The analysis of the seasonal distribution of viral pathogens showed that most outbreaks with low frequency viruses were restricted to a short period of time, which could be a result of a specific viral gene mutation that exploited the gap in population immunity, as typically seen with influenza viruses (Carter and Sanford, 2012; Reperant et al., 2015). In contrast, norovirus was detected in most months of the year; consistent with findings in multiple centers, which showed indiscriminately high frequencies of norovirus infections worldwide (Ahmed et al., 2014).

Among bacterial species we also found some interesting differences in seasonal distribution patterns. *V. parahaemolyticus* was only found from May to September contrasting with *Salmonella* detected in most months of the year. Since *V. parahaemolyticus* is often isolated from seawater and seafood, the consumption of contaminated seafood appears to be one of the leading causes of summer diarrheas in Shanghai and likely other coastal regions. On the other hand *Salmonella*, as an important foodborne pathogen with wide array of contamination sources accounting for its broad distribution (Campioni et al., 2012; Jackson et al., 2013).

In recent years, increasing antibiotic resistance of clinical isolates, especially MDR has been a major concern for human health, as it often results in prolonged illness and greater risk of death (Canton and Ruiz-Garbajosa, 2011). In this study, we analyzed the resistance profile of the diarrheal bacteria to 11 common antibiotics. All *V. parahaemolyticus* isolates showed a high level of resistance to cephalosporins (55.6–100.0%), contrary to the results of 2015 Shanghai study, which showed a low drug resistance rate to cephalosporins (Li et al., 2017). Also investigation of *V. parahaemolyticus* in southeastern China from 2009 to 2013 reported most isolates were sensitive to common antimicrobial agents apart from ampicillin (Chen et al., 2016). Thus, our results indicated the resistance rate to cephalosporins had markedly increased in Shanghai (Elmahdi et al., 2016). *Salmonella* exhibited high resistance rates to cefuroxime (CXM) and levofloxacin (LVX). However, CXM and LVX are still commonly used in treatment of acute infectious diarrhea. Remarkably, we found the MDR rates were high in all bacteria isolates, including *Shigella* which demonstrated 100% MDR frequency. The most common resistance combination of *V. parahaemolyticus* and *Salmonella* was cephalosporins and quinolone, which are also the most commonly used antibiotics in our region. The bacteria may acquire resistance to antibiotics via mutations in chromosomal genes or by horizontal gene transfer induced by excess antibiotic application. In addition, the spread of drug-resistant genes mediated by plasmids or bacteriophage may also be a potential cause (Blair et al., 2015; Lekunberri et al., 2017). While our study could not identify mechanisms by which the isolates acquired resistance, they showed that the drug resistance was widely and quite uniformly spread across multiple genotypic variants (**Table 3**), and thus no statistically significant association was found between resistance and ST strain classification. Together, our findings support that antibiotic resistance reached critical level and this needs to be addressed in clinical practice.

The final group of conclusions pertains to genetic diversity and distribution of common virulence genes critical for pathogenicity of *V. parahaemolyticus* and *Salmonella* across their different STs. This data set only marks the beginning of longitudinal studies that will help us to dissect trends pathogens’ genetic microevolution overtime. While, ST3 presented the most dominant ST in *V. parahaemolyticus*, consistent with previous study in Asian countries (Han et al., 2016), it strikingly differs from the predominant types in Maryland, United: ST631 and ST36 (Haendiges et al., 2015) not found in our study. This is not surprising since the origins of *V. parahaemolyticus* at both sites are linked to Pacific and Atlantic Ocean, respectively, and involve different continents. Future studies, especially from the American West Coast may provide further insights, if the divergence of ST type is linked more to the aquatic or continental location (local micro evolution) and also how this may be further affected by globalization of the seafood industry. The pandemic serotype of *V. parahaemolyticus* were O3:K6, consistent with the findings from other places worldwide (Ceccarelli et al., 2013; Velazquez-Roman et al., 2014). In terms of virulence, human *V. parahaemolyticus* isolates detected in this study contained *tlh* gene a hallmark of virulent *V. parahaemolyticus* (Banerjee et al., 2014; Chen et al., 2016) and almost equally and uniformly common across STs gene. The *ureR* gene and *trh* were both collectively absent. Since, *ureR* is located immediately upstream of *trh*, our results further support that while not necessary for virulence, these two virulence-associated genes are genetically linked, and complications introduced by inter-strains sequence heterogeneity might be avoided by detection of the *ureR* gene (Nilsson and Turner, 2016). Overall, conclusion is that *V. parahaemolyticus* is largely dominated by one or two STs and serotypes, the serotypes were generally associated with STs of *V. parahaemolyticus*, none apparent association between genetic diversities, serotypes with its resistance phenotypes was detected in our results, consistent with uniformly wide-spread resistance among all most prevalent ST types found in Shanghai.

Among the 23 *Salmonella* isolates, the most frequently detected was ST11, followed by ST19 and ST34 in contrast with reported predominant STs of *Salmonella* isolates were ST8, ST4 and ST3 in America (Dutta et al., 2014). These outcomes reveal important regional differences in Salmonella STs. Serovar Enteritidis was the most frequently isolated followed by Typhimurium, consistent with the data from foodborne outbreaks in America (Andino and Hanning, 2015). Regarding *Salmonella* virulence genes distribution, SPIs genes were present in almost all isolates, while SPV genes only occurred in 35–44% of the isolates, which demonstrated that the significant variations in pathogenicity among isolates were mainly attributed to the spread of plasmid genes. Furthermore, genetic distribution of virulence factors appears to show stronger link with STs compared with *V. parahaemolyticus*, consistent with previous reports linking STs with virulence and resistance (Luo et al., 2014; Li et al., 2017). In our study, most ST19 *Salmonella* isolates were positive for SPV and SPI genes, corresponding patients’ information showing more severe symptoms in ST19-infected patients. This was in contrast with *Salmonella typhimurium*, which was corresponding to ST19 and ST34 isolates. Meanwhile, all other STs except for ST11 were negative for all SPV genes but all SPI genes, similar to serovars Newport, Thompson, Montevideo, London, Grampian and Give. Finally, the very broad resistance to multiple antibiotics was uniformly spread across various STs and serotypes indicating that the rate of acquiescence of drug resistance is very fast and occurs independent of STs and serotypes. Based on these findings, we recommended the further longitudinal and multi-center studies monitoring the virulence along with genotype of ST classification.

## Conclusion

Our study systematically explored the distribution, seasonality, STs, serotypes, antimicrobial susceptibility phenotype and distribution of virulence genes of the pathogens from patients with acute diarrhea in Shanghai in 2016. The results highlighted the regional difference of diarrhea pathogens, confirmed that *V. parahaemolyticus, Salmonella* and norovirus still presented as the most prevalent pathogens in the region, exhibited the main STs, serotypes, emphasized the serious antibiotic resistance, explored the association of virulence with genetic diversity and serotypes of *V. parahaemolyticus* and *Salmonella* isolates. This study would provide useful information for comparing of molecular epidemiology in our region over time and among different regions to guide appropriate therapeutic strategies and monitoring the variation of genotypes of pathogens causing acute diarrheas.

## Author Contributions

FY, YJ, LY, and JQ contributed to this work equally. CY, MC, YMZ, and HuZ designed and coordinated the study. FY, YJ, LY, JQ, and MG collected specimens and detected the pathogens. YL, HC, YZ, and JZ designed the primers and did the data analysis. FY, ML, HoZ, ZD, YMZ, and HuZ interpreted the data and prepared the manuscript. All the authors approved the submission of the manuscript.

## Conflict of Interest Statement

The authors declare that the research was conducted in the absence of any commercial or financial relationships that could be construed as a potential conflict of interest.

## Footnotes

1 http://pubmlst.org

2 http://mlst.warwick.ac.uk/mlst/

## References

[B1] Achtman M. Wain J. Weill F. X. Nair S. Zhou Z. Sangal V. (2012). Multilocus sequence typing as a replacement for serotyping in *Salmonella enterica*. *PLOS Pathog.* 8:e1002776. 10.1371/journal.ppat.1002776 22737074 PMC3380943

[B2] Ahmed S. M. Hall A. J. Robinson A. E. Verhoef L. Premkumar P. Parashar U. D. (2014). Global prevalence of norovirus in cases of gastroenteritis: a systematic review and meta-analysis. *Lancet Infect. Dis.* 14 725–730. 10.1016/S1473-3099(14)70767-4 24981041 PMC8006533

[B3] Aly M. Wiltshire S. Chahrour G. Osti J. C. Vidal S. M. (2007). Complex genetic control of host susceptibility to coxsackievirus B3-induced myocarditis. *Genes Immun.* 8 193–204. 10.1038/sj.gene.6364374 17287827

[B4] Andino A. Hanning I. (2015). Salmonella enterica: survival, colonization, and virulence differences among serovars. *ScientificWorldJournal* 2015:520179. 10.1155/2015/520179 25664339 PMC4310208

[B5] Arias C. A. Murray B. E. (2009). Antibiotic-resistant bugs in the 21st century–a clinical super-challenge. *N. Engl. J. Med.* 360 439–443. 10.1056/NEJMp0804651 19179312

[B6] Atmar R. L. Bernstein D. I. Harro C. D. Al-Ibrahim M. S. Chen W. H. Ferreira J. (2011). Norovirus vaccine against experimental human Norwalk Virus illness. *N. Engl. J. Med.* 365 2178–2187. 10.1056/NEJMoa1101245 22150036 PMC3761795

[B7] Banerjee S. K. Kearney A. K. Nadon C. A. Peterson C. L. Tyler K. Bakouche L. (2014). Phenotypic and genotypic characterization of Canadian clinical isolates of *Vibrio parahaemolyticus* collected from 2000 to 2009. *J. Clin. Microbiol.* 52 1081–1088. 10.1128/JCM.03047-13 24452166 PMC3993483

[B8] Blair J. M. Webber M. A. Baylay A. J. Ogbolu D. O. Piddock L. J. (2015). Molecular mechanisms of antibiotic resistance. *Nat. Rev. Microbiol.* 13 42–51. 10.1038/nrmicro3380 25435309

[B9] Campioni F. Moratto Bergamini A. M. Falcao J. P. (2012). Genetic diversity, virulence genes and antimicrobial resistance of *Salmonella* Enteritidis isolated from food and humans over a 24-year period in Brazil. *Food Microbiol.* 32 254–264. 10.1016/j.fm.2012.06.008 22986188

[B10] Canton R. Ruiz-Garbajosa P. (2011). Co-resistance: an opportunity for the bacteria and resistance genes. *Curr. Opin. Pharmacol.* 11 477–485. 10.1016/j.coph.2011.07.007 21840259

[B11] Carter R. W. Sanford J. C. (2012). A new look at an old virus: patterns of mutation accumulation in the human H1N1 influenza virus since 1918. *Theor. Biol. Med. Model.* 9:42. 10.1186/1742-4682-9-42 23062055 PMC3507676

[B12] Ceccarelli D. Hasan N. A. Huq A. Colwell R. R. (2013). Distribution and dynamics of epidemic and pandemic *Vibrio parahaemolyticus* virulence factors. *Front. Cell Infect. Microbiol.* 3:97 10.3389/fcimb.2013.00097 PMC3858888 24377090

[B13] Chen Y. Chen X. Yu F. Wu M. Wang R. Zheng S. (2016). Serology, virulence, antimicrobial susceptibility and molecular characteristics of clinical *Vibrio parahaemolyticus* strains circulating in southeastern China from 2009 to 2013. *Clin. Microbiol. Infect.* 22 258 e9–16. 10.1016/j.cmi.2015.11.003 26597222

[B14] Chi C. Y. Liao L. N. Ho C. M. Chou C. H. Ho M. W. Wang J. H. (2017). Epidemiology, clinical features, and microbiology of patients with diarrhea in community clinics in Taiwan. *J. Microbiol. Immunol. Infect.* 10.1016/j.jmii.2017.05.0031 [Epub ahead of print]. 28688828

[B15] Das J. K. Bhutta Z. A. (2016). Global challenges in acute diarrhea. *Curr. Opin. Gastroenterol.* 32 18–23. 10.1097/MOG.0000000000000236 26574867

[B16] Denamur E. Matic I. (2006). Evolution of mutation rates in bacteria. *Mol. Microbiol.* 60 820–827. 10.1111/j.1365-2958.2006.05150.x 16677295

[B17] Dutta S. Das S. Mitra U. Jain P. Roy I. Ganguly S. S. (2014). Antimicrobial resistance, virulence profiles and molecular subtypes of *Salmonella enterica* serovars Typhi and Paratyphi A blood isolates from Kolkata, India during 2009-2013. *PLOS ONE* 9:e101347. 10.1371/journal.pone.0101347 25098613 PMC4123848

[B18] Elmahdi S. Dasilva L. V. Parveen S. (2016). Antibiotic resistance of *Vibrio parahaemolyticus* and *Vibrio vulnificus* in various countries: a review. *Food Microbiol.* 57 128–134. 10.1016/j.fm.2016.02.008 27052711

[B19] Estevez A. Arvelo W. Hall A. J. Lopez M. R. Lopez B. Reyes L. (2013). Prevalence and genetic diversity of norovirus among patients with acute diarrhea in Guatemala. *J. Med. Virol.* 85 1293–1298. 10.1002/jmv.23578 23595770 PMC4664073

[B20] Farthing M. Salam M. A. Lindberg G. Dite P. Khalif I. Salazar-Lindo E. (2013). Acute diarrhea in adults and children: a global perspective. *J. Clin. Gastroenterol.* 47 12–20. 10.1097/MCG.0b013e31826df662 23222211

[B21] GBD 2015 Mortality and Causes of Death Collaborators (2016). Global, regional, and national life expectancy, all-cause mortality, and cause-specific mortality for 249 causes of death, 1980-2015: a systematic analysis for the Global Burden of Disease Study 2015. *Lancet* 388 1459–1544. 10.1016/S0140-6736(16)31012-1 27733281 PMC5388903

[B22] Gong J. Kelly P. Wang C. (2017). Prevalence and antimicrobial resistance of *Salmonella enterica* serovar Indiana in China (1984-2016). *Zoonoses Public Health* 64 239–251. 10.1111/zph.12328 28009105

[B23] Groisman E. A. Ochman H. (1996). Pathogenicity islands: bacterial evolution in quantum leaps. *Cell* 87 791–794. 10.1016/S0092-8674(00)81985-6 8945505

[B24] Groisman E. A. Ochman H. (1997). How *Salmonella* became a pathogen. *Trends Microbiol.* 5 343–349. 10.1016/S0966-842X(97)01099-8 9294889

[B25] Guerrant R. L. Kosek M. Moore S. Lorntz B. Brantley R. Lima A. A. (2002). Magnitude and impact of diarrheal diseases. *Arch. Med. Res.* 33 351–355. 10.1016/S0188-4409(02)00379-X 12234524

[B26] Haendiges J. Timme R. Allard M. W. Myers R. A. Brown E. W. Gonzalez-Escalona N. (2015). Characterization of *Vibrio parahaemolyticus* clinical strains from Maryland (2012-2013) and comparisons to a locally and globally diverse *V. parahaemolyticus* strains by whole-genome sequence analysis. *Front. Microbiol.* 6:125. 10.3389/fmicb.2015.00125 25745421 PMC4333860

[B27] Han C. Tang H. Ren C. Zhu X. Han D. (2016). Sero-prevalence and genetic diversity of pandemic *V. parahaemolyticus* strains occurring at a global scale. *Front. Microbiol.* 7:567. 10.3389/fmicb.2016.00567 27148244 PMC4840284

[B28] He X. Xie M. Li S. Ye J. Peng Q. Ma Q. (2017). Antimicrobial resistance in bacterial pathogens among hospitalized children with community acquired lower respiratory tract infections in Dongguan, China (2011-2016). *BMC Infect. Dis.* 17:614. 10.1186/s12879-017-2710-4 28893195 PMC5594502

[B29] Hilmarsdottir I. Baldvinsdottir G. E. Harethardottir H. Briem H. Sigurethsson S. I. (2012). Enteropathogens in acute diarrhea: a general practice-based study in a Nordic country. *Eur. J. Clin. Microbiol. Infect. Dis.* 31 1501–1509. 10.1007/s10096-011-1470-0 22057365

[B30] Hu L. F. Xu X. H. Li H. R. Gao L. P. Chen X. Sun N. (2017). Surveillance of antimicrobial susceptibility patterns among *Stenotrophomonas maltophilia* isolated in China during the 10-year period of 2005-2014. *J. Chemother.* 30 25–30. 10.1080/1120009X.2017.1378834 28949279

[B31] Huhulescu S. Kiss R. Brettlecker M. Cerny R. J. Hess C. Wewalka G. (2009). Etiology of acute gastroenteritis in three sentinel general practices, Austria 2007. *Infection* 37 103–108. 10.1007/s15010-008-8106-z 19148576

[B32] Jackson B. R. Griffin P. M. Cole D. Walsh K. A. Chai S. J. (2013). Outbreak-associated *Salmonella enterica* serotypes and food Commodities, United States, 1998-2008. *Emerg. Infect. Dis.* 19 1239–1244. 10.3201/eid1908.121511 23876503 PMC3739514

[B33] Jia L. Lin C. Gao Z. Qu M. Yang J. Sun J. (2016). Prevalence and factors associated with different pathogens of acute diarrhea in adults in Beijing, China. *J. Infect. Dev. Ctries.* 10 1200–1207. 10.3855/jidc.6831 27886032

[B34] Jones J. L. Ludeke C. H. Bowers J. C. Garrett N. Fischer M. Parsons M. B. (2012). Biochemical, serological, and virulence characterization of clinical and oyster *Vibrio parahaemolyticus* isolates. *J. Clin. Microbiol.* 50 2343–2352. 10.1128/JCM.00196-12 22535979 PMC3405591

[B35] Jorgensen J. H. Pfaller M. A. (2015). *Manual of Clinical Microbiology*. Washington, DC: ASM press.

[B36] Kabir M. R. Hossain M. A. Paul S. K. Mahmud C. Ahmad S. Mahmud N. U. (2012). Enteropathogens associated with acute diarrhea in a tertiary hospital of Bangladesh. *Mymensingh Med. J.* 21 618–623. 23134907

[B37] Kim J. K. Kim J. W. (2014). Molecular epidemiologic trends of diarrhea-causing virus infection from clinical specimens in Cheonan, Korea, in 2010-2012. *J. Clin. Lab. Anal.* 28 47–51. 10.1002/jcla.21642 24375926 PMC6807402

[B38] Lekunberri I. Subirats J. Borrego C. M. Balcazar J. L. (2017). Exploring the contribution of bacteriophages to antibiotic resistance. *Environ. Pollut.* 220 981–984. 10.1016/j.envpol.2016.11.059 27890586

[B39] Li H. Tang R. Lou Y. Cui Z. Chen W. Hong Q. (2017). A comprehensive epidemiological research for clinical *Vibrio parahaemolyticus* in Shanghai. *Front. Microbiol.* 8:1043 10.3389/fmicb.2017.01043 PMC5462930 28642752

[B40] Lozano R. Naghavi M. Foreman K. Lim S. Shibuya K. Aboyans V. (2012). Global and regional mortality from 235 causes of death for 20 age groups in 1990 and 2010: a systematic analysis for the Global Burden of Disease Study 2010. *Lancet* 380 2095–2128. 10.1016/S0140-6736(12)61728-0 23245604 PMC10790329

[B41] Luo Y. Wang Y. Ye L. Yang J. (2014). Molecular epidemiology and virulence factors of pyogenic liver abscess causing *Klebsiella pneumoniae* in China. *Clin. Microbiol. Infect.* 20 O818–O824. 10.1111/1469-0691.12664 24804560

[B42] Magiorakos A. P. Srinivasan A. Carey R. B. Carmeli Y. Falagas M. E. Giske C. G. (2012). Multidrug-resistant, extensively drug-resistant and pandrug-resistant bacteria: an international expert proposal for interim standard definitions for acquired resistance. *Clin. Microbiol. Infect.* 18 268–281. 10.1111/j.1469-0691.2011.03570.x 21793988

[B43] Mo Q. H. Wang H. B. Dai H. R. Lin J. C. Tan H. Wang Q. (2015). Rapid and simultaneous detection of three major diarrhea-causing viruses by multiplex real-time nucleic acid sequence-based amplification. *Arch. Virol.* 160 719–725. 10.1007/s00705-014-2328-4 25559674

[B44] Nilsson W. B. Turner J. W. (2016). The thermostable direct hemolysin-related hemolysin (trh) gene of *Vibrio parahaemolyticus*: sequence variation and implications for detection and function. *J. Microbiol. Methods* 126 1–7. 10.1016/j.mimet.2016.04.007 27094247

[B45] Patel M. M. Hall A. J. Vinje J. Parashar U. D. (2009). Noroviruses: a comprehensive review. *J. Clin. Virol.* 44 1–8. 10.1016/j.jcv.2008.10.009 19084472

[B46] Reperant L. A. Grenfell B. T. Osterhaus A. D. (2015). Quantifying the risk of pandemic influenza virus evolution by mutation and re-assortment. *Vaccine* 33 6955–6966. 10.1016/j.vaccine.2015.10.056 26603954

[B47] Riddle M. S. Dupont H. L. Connor B. A. (2016). ACG clinical guideline: diagnosis, treatment, and prevention of acute diarrheal infections in adults. *Am. J. Gastroenterol.* 111 602–622. 10.1038/ajg.2016.126 27068718

[B48] Scallan E. Hoekstra R. M. Angulo F. J. Tauxe R. V. Widdowson M. A. Roy S. L. (2011). Foodborne illness acquired in the United States–major pathogens. *Emerg. Infect. Dis.* 17 7–15. 10.3201/eid1701.11101 21192848 PMC3375761

[B49] Swierczewski B. E. Odundo E. A. Koech M. C. Ndonye J. N. Kirera R. K. Odhiambo C. P. (2013). Surveillance for enteric pathogens in a case-control study of acute diarrhea in Western Kenya. *Trans. R. Soc. Trop. Med. Hyg.* 107 83–90. 10.1093/trstmh/trs022 23222955

[B50] Taniguchi H. Hirano H. Kubomura S. Higashi K. Mizuguchi Y. (1986). Comparison of the nucleotide sequences of the genes for the thermostable direct hemolysin and the thermolabile hemolysin from *Vibrio parahaemolyticus*. *Microb. Pathog.* 1 425–432. 10.1016/0882-4010(86)90004-5 3508495

[B51] Velazquez-Roman J. Leon-Sicairos N. De Jesus Hernandez-Diaz L. Canizalez-Roman A. (2014). Pandemic *Vibrio parahaemolyticus* O3:K6 on the American continent. *Front. Cell Infect. Microbiol.* 3:110. 10.3389/fcimb.2013.00110 24427744 PMC3878053

[B52] Vidovic S. Aly M. Flemming C. Springthorpe S. Sattar S. A. (2011). First evidence of genotypes Ad3a16 and Ad3a18 in North America, obtained by genetic analysis of infectious human adenovirus from wastewaters of two urban communities in Canada. *Appl. Environ. Microbiol.* 77 4256–4259. 10.1128/AEM.02795-10 21515731 PMC3131634

[B53] Wu S. Wu Q. Zhang J. Chen M. Guo W. (2016). Analysis of multilocus sequence typing and virulence characterization of *Listeria monocytogenes* isolates from Chinese retail ready-to-eat food. *Front. Microbiol.* 7:168. 10.3389/fmicb.2016.00168 26909076 PMC4754575

[B54] Yang Y. Chen J. Lin D. Xu X. Cheng J. Sun C. (2017). Prevalence and drug resistance characteristics of carbapenem-resistant *Enterobacteriaceae* in Hangzhou, China. *Front. Med.* 10.1007/s11684-017-0529-4 [Epub ahead of print]. 28687975

[B55] Zhang Y. Zhao Y. Ding K. Wang X. Chen X. Liu Y. (2014). Analysis of bacterial pathogens causing acute diarrhea on the basis of sentinel surveillance in Shanghai, China, 2006-2011. *Jpn. J. Infect. Dis.* 67 264–268. 10.7883/yoken.67.264 25056071

[B56] Zhu M. Zhang J. Gao Y. Wang Z. (2008). [Present research on disease burden of diarrhea illness]. *Wei Sheng Yan Jiu* 37 126–128. 18421885

