# Supplementary material for: Molecular and Conventional Analysis of Acute Diarrheal Isolates Identifies Epidemiological Trends, Antibiotic Resistance and Virulence Profiles of Common Enteropathogens in Shanghai
Source: Front Microbiol. 2018 Feb 9;9:164. doi: 10.3389/fmicb.2018.00164 (PMC5845389; doi:10.3389/fmicb.2018.00164)
Supplement: Supplementary file 1 [file Table_1.DOCX]

| **Origin** | **Gene** | **Forward primers (5’-3’)** | **Reverse primers (5’-3’)** | **Size (bp)** |
| --- | --- | --- | --- | --- |
| ***V. parahaemolyticus*^a^** |  |  |  |  |
|  | *recA* | tgtaaaacgacggccagtGAAACCATTTCAACGGGTTC | caggaaacagctatgaccCCATTGTAGCTGTACCAAGCACCC | 773 |
|  | *gyrB* | tgtaaaacgacggccagtGAAGGBGGTATTCAAGC | caggaaacagctatgaccGAGTCACCCTCCACWATGTA | 629 |
|  | *dnaE* | tgtaaaacgacggccagtCGRATMACCGCTTTCGCCG | caggaaacagctatgaccGAKATGTGTGAGCTGTTTGC | 596 |
|  | *dtdS* | tgtaaaacgacggccagtTGGCCATAACGACATTCTGA | caggaaacagctatgaccGAGCACCAACGTGTTTAGC | 497 |
|  | *pntA* | tgtaaaacgacggccagtACGGCTACGCAAAAGAAATG | caggaaacagctatgaccTTGAGGCTGAGCCGATACTT | 470 |
|  | *pyrC* | tgtaaaacgacggccagtAGCAACCGGTAAAATTGTCG | caggaaacagctatgaccCAGTGTAAGAACCGGCACAA | 533 |
|  | *tnaA* | tgtaaaacgacggccagtTGTACGAAATTGCCACCAAA | caggaaacagctatgaccAATATTTTCGCCGCATCAAC | 463 |
| ***Salmonella***^b^ |  |  |  |  |
|  | *thrA* | GTCACGGTGATCGATCCGGT | CACGATATTGATATTAGCCCG | 852 |
|  | *purE* | GACACCTCAAAAGCAGCGT | AGACGGCGATACCCAGCGG | 510 |
|  | *sucA* | CGCGCTCAAACAGACCTAC | GACGTGGAAAATCGGCGCC | 643 |
|  | *hisD* | GAAACGTTCCATTCCGCGC | GCGGATTCCGGCGACCAG | 894 |
|  | *aroC* | CCTGGCACCTCGCGCTATAC | CCACACACGGATCGTGGCG | 826 |
|  | *hemD* | GAAGCGTTAGTGAGCCGTCTGCG | ATCAGCGACCTTAATATCTTGCCA | 666 |
|  | *dnaN* | ATGAAATTTACCGTTGAACGTGA | AATTTCTCATTCGAGAGGATTGC | 833 |
| ***Salmonella***^c^ |  |  |  |  |
|  | *thrA* | ATCCCGGCCGATCACATGAT | ACCGCCAGCGGCTCCAGCA | 501 |
|  | *purE* | ACAGGAGTTTTAAGACGCATG | GCAAACTTGCTTCATAGCG | 399 |
|  | *sucA* | CCGAAGAGAAACGCTGGATC | GGTTGTTGATAACGATACGTAC | 501 |
|  | *hisD* | GTCGGTCTGTATATTCCCGG | GGTAATCGCATCCACCAAATC | 501 |
|  | *aroC* | GGCGTGACGACCGGCAC | AGCGCCATATGCGCCAC | 501 |
|  | *hemD* | GCCTGGAGTTTTCCACTG | GACCAATAGCCGACAGCGTAG | 432 |
|  | *dnaN* | CCGATTCTCGGTAACCTGCT | ACGCGACGGTAATCCGGG | 501 |
| ***V. parahaemolyticus*^d^** |  |  |  |  |
|  | *tlh* | AAAGCGGATTATGCAGAAGCACTG | GCTACTTTCTAGCATTTTCTCTGC | 450 |
|  | *tdh* | GTAAAGGTCTCTGACTTTGGAC | TGGAATAGAACCTTCATCTTCACC | 270 |
|  | *trh* | TTGGCTTCGATATTTTCAGTATCT | CATAACAAACATATGCCCATTTCCG | 500 |
|  | *ureR* | ATGGAATACAAAAACATTCAATCATC | ATTATCTGAAAGTTACGTTCAACGC | 350 |
| ***Salmonella***^e^ |  |  |  |  |
|  | *invA* | ACAGTGCTCGTTTACGACCTGAAT | AGACGACTGGTACTGATCGATAAT | 500 |
|  | *hilA* | CCTTACGACGTATTCTGTCGG | GCGATAATCCCTTCACGATA | 207 |
|  | *ssaQ* | GAATAGCGAATGAAGAGCG | TCCCATCGTGTTATCCTCTGTCAGC | 677 |
|  | *ssrB* | GGTCTTGAGGTTTATAATGC | CTGTCGTGTCAGCGTTTAATTCA | 322 |
|  | *spiA* | CCAGGGGTCGTTAGTGTATTGCGTGAGATG | CGCGTAACAAAGAACCCGTAGTGATGGATT | 550 |
|  | *mgtC* | AAAGACAATGGCGTCAACGTATGG | TTCTTTATAGCCCTGTTCCTGAGC | 500 |
|  | *orfL* | GGAGTATCGATAAAGATGTT | GCGCGTAACGTCAGAATCAA | 332 |
|  | *sopB* | ATGCTCAGGTCAAGCAGC | ACCGTGGACATCCACAAA | 214 |
|  | *safB* | TCCGTACTGGTGGTGACAT | GGGATATACTCAAGCCCTGTAA | 316 |
|  | *spvA* | GCTGACCGGCGATAACAGTA | AATCGCTAACTGTCGGGCAA | 196 |
|  | *spvB* | CTATCAGCCCCGCACGGAGAGCAGTTTTTA | GGAGGAGGCGGTGGCGGTGGCATCATA | 717 |
|  | *spvC* | GACTATTCAGGGATGCGCCA | TGCCGTGTCTTGTGGAGAAA | 356 |
| **16S rDNA** | | ATATATTTGAGAGGCATCTCTTGA | CTGCACTCAAGTCTCCCAGTT | 456 |

**Supplementary table 1.** Genes and primers used in this study

^a^ The amplification primers of MLST for *V. parahaemolyticus*, which contain attached M13 forward and reverse primers; the sequencing was conducted using M13 primers (forward and reverse). ^b^ The amplification primers of MLST for *Salmonella*. ^c^ The sequencing primers of MLST for *Salmonella*. ^d^ Primers for detection of virulence genes of *V. parahaemolyticus*. ^e^ Primers for detection of virulence genes of *Salmonella*.
